# Pharmacophore-Based Discovery of Substrates of a Novel Drug/Proton-Antiporter in the Human Brain Endothelial hCMEC/D3 Cell Line

**DOI:** 10.3390/pharmaceutics14020255

**Published:** 2022-01-21

**Authors:** Maria Smirnova, Laura Goracci, Gabriele Cruciani, Laetitia Federici, Xavier Declèves, Hélène Chapy, Salvatore Cisternino

**Affiliations:** 1 Université de Paris, INSERM UMR_S1144, Optimisation Thérapeutique en Neuropsychopharmacologie, 75006 Paris, France; maria.smirnova@inserm.fr (M.S.); laetitia.federici@etu.u-paris.fr (L.F.); xavier.decleves@u-paris.fr (X.D.); helene.chapy@etu.parisdescartes.fr (H.C.); 2 Biology and Biotechnology, Department of Chemistry, University of Perugia, 06123 Perugia, Italy; laura.goracci@unipg.it (L.G.); gabriele.cruciani@unipg.it (G.C.); 3 Biologie du Médicament et Toxicologie, AP-HP, Hôpital Cochin, 75014 Paris, France; 4 Service Pharmacie, AP-HP, Hôpital Necker-Enfants Malades, 75015 Paris, France

**Keywords:** blood-brain barrier, biological transport, computational chemistry, drug delivery, FLAPpharm approach, in silico, pharmacophore-based substrate discovery

## Abstract

A drug/proton-antiporter, whose the molecular structure is still unknown, was previously evidenced at the blood-brain barrier (BBB) by functional experiments. The computational method could help in the identification of substrates of this solute carrier (SLC) transporter. Two pharmacophore models for substrates of this transporter using the FLAPpharm approach were developed. The trans-stimulation potency of 40 selected compounds for already known specific substrates ([^3^H]-clonidine) were determined and compared in the human brain endothelial cell line hCMEC/D3. Results. The two pharmacophore models obtained were used as templates to screen xenobiotic and endogenous compounds from four databases (e.g., Specs), and 45 hypothetical new candidates were tested to determine their substrate capacity. Psychoactive drugs such as antidepressants (e.g., imipramine, desipramine), antipsychotics/neuroleptics such as phenothiazine derivatives (chlorpromazine), sedatives anti-histamine-H_1_ drugs (promazine, promethazine, triprolidine, pheniramine), opiates/opioids (e.g., hydrocodone), trihexyphenidyl and sibutramine were correctly predicted as proton-antiporter substrates. The best performing pharmacophore model for the proton-antiporter substrates appeared as a good predictor of known substrates and allowed the identification of new substrate compounds. This model marks a new step in the characterization of this drug/proton-antiporter and will be of great use in uncovering its substrates and designing chemical entities with an improved influx capability to cross the BBB.

## 1. Introduction

The blood-brain barrier (BBB), formed by the endothelial cells of brain capillaries, is known to efficiently restrict the brain access of many compounds, making it a challenge to develop new pharmacological agents targeting the central nervous system (CNS) [1]. Since the early discovery of tight junctions linking brain endothelial cells to each other and impeding the BBB paracellular route, more recent biochemical insights have better illustrated the role played by xenobiotic transporters in the control of the drug access to the brain parenchyma as well as in many body interfaces such as the intestine. This knowledge has advanced our understanding of the dynamics and fate of drugs across membranes and cellular interfaces.

The ATP-binding cassette proteins (ABC) P-glycoprotein (P-gp/ABCB1), breast cancer resistance protein (BCRP/ABCG2), and multidrug resistance associated protein 4 (MRP4/ABCC4) are the main ABC transporters known to limit brain drug distribution at the BBB [1,2]. They only mediate the unidirectional brain exit of substrates across the luminal BBB membrane back to the blood compartment, whatever the direction of the substrate concentration-gradient flux. The ability of psychoactive drugs to permeate the BBB has therefore been pictured as an absence of their transport by these ABC proteins and their ability to passively diffuse through the BBB. Based on these “all passive diffusion” assumptions, efforts have been made to predict the permeability of CNS drugs using physicochemical descriptors for passive membrane diffusion of the unionized/neutral drug, also known as the drug pH-partition hypothesis. Interestingly, many psychoactive drugs are predominantly basic and cationic at physiological pH.

Recent discoveries of solute carriers (SLC) for organic cations, such as the OCT1-3 (SLC22A1-3), and MATE1 (SLC47A1) transporters, have greatly improved our comprehension of their critical and determinant role in the absorption, distribution, and/or elimination of drugs in the body [3,4,5]. SLC transporters, unlike ABC drug transporters, are mainly bidirectional, and their net flux of transport (i.e., influx or efflux) will match the most favorable thermodynamic force-flux of their organic or inorganic substrate. However, there is still a critical need for deorphanization of transporters of the SLC superfamily, since many transporters for drugs or solutes have been functionally characterized without their molecular identity/structure being elucidated to date [6,7].

Indeed, studies in several in vivo (mouse/rat BBB) and in vitro models (rodent and human brain endothelial cells) have provided functional evidence for a SLC drug proton-antiporter with unknown structure that controls the BBB transport of some CNS compounds [8,9,10,11,12,13,14,15,16]. Clonidine, an α_2_-adrenergic CNS-acting agonist that is clinically valuable as an antihypertensive, is a prototypical and specific substrate used for the characterization of this transporter, as previously shown at the mouse BBB [8] and in the human brain endothelial cell line hCMEC/D3 [9]. So far, the identified substrates include mainly cationic tertiary/secondary amines such as cocaine, clonidine, naloxone, oxycodone, ecstasy and diphenhydramine, which were used in many studies to illustrate some function properties of this drug/proton-antiporter [8,10,11,12,13,17]. For these CNS drugs, the BBB transport by this drug/proton-antiporter dominates their brain passive diffusion and may represent a critical determinant of their CNS effects. This recent knowledge has definitively reshaped the concept of BBB permeability for CNS drugs as a possible effect from multiple mechanisms that could simultaneously involve molecular fluxes from passive diffusion, ABC and/or SLC working together even in opposition [18].

The features of this SLC drug proton-antiporter at the BBB highlight its importance in governing at least drug delivery to the brain. Further studies have also illustrated its function in other key body interfaces such as the intestine and retina [18,19,20,21,22]. Understanding its molecular interaction features by developing structure-activity relationship (SAR) models could help to predict drug interactions and make clearer its putative role in the pharmacokinetics (PK) and also in brain pharmacodynamics (PD) of its substrates.

We previously developed a pharmacophore model for inhibitors of this drug/proton-antiporter in the human brain endothelial cell line hCMEC/D3 [9] using the FLAPpharm algorithm and cis-inhibition experiments of prototypical substrates (e.g., [^3^H]-clonidine) [23]. FLAPpharm makes use of the GRID Molecular Interaction Fields (MIFs) of each selected active ligand to drive the alignment and search for the best superimposition. The resulting alignment model represents the base to derive a common pharmacophore, which can be also used as a template in virtual screening using the FLAP software [24,25]. In general, when pharmacophoric approaches are attempted, the diversity of the active ligands used for pharmacophore generation rather than their number plays a key role and this is also true when FLAPpharm is used [9]. Another important point of developing a pharmacophore is conformer generation for compounds to be aligned. In FLAPpharm, a stochastic approach based on the MM3-like forcefield is used to perform a random search using 40 samples for each rotatable bond. Thus, thousands of potential conformers are minimized, and an energy filter is applied to discard those conformers whose energy is greater than 20 kcal/mol above the minimum. Finally, the remaining conformers are ranked by energy criterion and filtered by applying a threshold root mean square difference (rmsd) to another lower energy conformer [23]. Validation results for the conformer generator within FLAPpharm have been extensively described elsewhere [23], and showed that the method assures conformer diversity and also the capability of including a conformation close to the crystallographic one in ~90% of cases. Once conformers are generated, the molecular interaction fields are calculated for each conformer using the H, O, N1, and DRY probes to take into account shape and hydrophobic, hydrogen-bond donor and hydrogen-bond acceptor interactions; thus, the FLAP fingerprints are generated and can be used to drive alignment seeking for the optimal MIF similarity. Obtained pharmacophore models are scored using a parameterized scoring function, which is a weighted sum of MIF similarities.

To determine the substrate capacity of candidates for the drug/proton-antiporter the trans-stimulation effect of the specific substrate ([^3^H]-clonidine) was measured in hCMEC/D3 cells. Trans-stimulation is a known biochemical property exhibited by many SLC transporters and previously successfully used to identify SLC transporter substrates without the need of quantifying their cellular concentrations [8,11,26,27,28]. This property is based on the reorientation of the transporter substrate site between the two faces of the membrane that occurs more rapidly when it is occupied by a substrate than when it is empty [26]. Basically, this property means that the drug/proton-antiporter will mediate the exit of the intracellular loaded [^3^H]-clonidine greater when a substrate is present on the cytosolic face of the membrane, but will be unaffected by the presence of compound that does not interact as a substrate of this clonidine/drug/proton-antiporter.

In the present work we developed and validated new pharmacophore models for substrates of this drug/proton-antiporter using FLAPpharm. Indeed, although many inhibitors are also substrates, it is known that this relationship is not always true. Thus, the chemical features of a pharmacophore for substrates could differ from those of a pharmacophore for inhibitors. To validate substrate pharmacophores, two models were generated and corroborated using a series of compounds with known substrate or non-substrate properties, and used to identify novel substrates through virtual screening campaigns. A number of hypothetical hit compounds were identified and evaluated in hCMEC/D3 cells to show that a substrate pharmacophore generated by selecting compounds to be aligned through an unbiased principal component analysis (PCA) model was the most effective in predicting substrate candidates. This FLAPpharm approach [23] used here for pharmacophore generation is likely to be of great use for the discovery and design of new chemical compounds targeting the CNS and possibly other proton-antiporter-expressing tissues.

## 2. Materials and Methods

### 2.1. Drugs and Chemicals

[^3^H]-Clonidine (61.3 Ci/mmol) was purchased from Perkin Elmer (Courtaboeuf, France). Specs compounds (AE; AF; AG; AI; AK; AN; AO; AP) were purchased from Specs (Delft, The Netherlands). 6-monoacetylmorphine, codeine, cocaethylene, norcocaine, desomorphine, dihydromorphine, hydrocodone, hydromorphone, norbuprenorphine, oxycodone, and oxymorphone were obtained from Lipomed (Euromedex, Souffelweyersheim, France) and heroin, methadone, and morphine from Francopia (Paris, France). Methylenedioxy-methylamphetamine (MDMA, ecstasy) was synthetized by Dr H. Galons from the chemistry department of Paris University. All other chemicals were purchased from Sigma (St Quentin Fallavier, France) except diphenhydramine (Inresa, Strasbourg, France), cocaine (Cooper, Melun, France), and D617 (Carbosynth, Campton, UK).

### 2.2. Cell Culture

hCMEC/D3 cells were seeded on T-flask coated with type I collagen (25,000 cells/cm^2^) in EBM-2 medium (Lonza, Basel, Switzerland) supplemented with hydrocortisone, ascorbic acid, basic FGF (Sigma, St Quentin, France), fetal bovine serum (PAA, Pashing Austria) and HEPES (Invitrogen, Cergy-Pontoise, France), and grown at 37 °C under humidified atmosphere of 95% O_2_/5% CO_2_. For experiments, cells seeded in 24 multiwell dishes (25,000 cells/cm^2^) were used 3–4 days after seeding and between passages 28–35.

### 2.3. In Vitro [^3^H]-Clonidine Trans-Stimulation Experiments in hCMEC/D3 Cells for Substrate Screening

hCMEC/D3 cells cultured as describe above were first pre-incubated 30 min with Krebs-HEPES (KH) incubation buffer (128 mM NaCl, 24 mM NaHCO_3_, 4.2 mM KCl, 2.4 mM NaH_2_PO_4_, 1.5 mM CaCl_2_, 0.9 mM MgSO_4_, HEPES 10 mM, and 9 mM D-glucose). The pH of the incubation buffer was monitored and adjusted to 7.40. Trans-stimulation experiments were performed as previously reported elsewhere to identify substrates [8,11,26,27,28,29]. Briefly, the trans-stimulation assay requires first the incubation/loading of the specific tracer probe substrate ([^3^H]-clonidine) into the cell, and secondly the loaded cells are incubated with the candidate compound in a tracer/probe-free medium. The drug/proton-antiporter-mediated transport of the candidate/substrate compound inside the cells will more quickly relocate the binding site into the cell interior (or “trans-compartment”), and will be associated with an increase in the exit out of the cell of the tracer probe substrate (in this study [^3^H]-clonidine).

Accordingly, cells were first incubated for 5 min at 37 °C with KH buffer containing [^3^H]-clonidine (3.7 kBq/mL, ~2 nM), a known validated specific substrate of the proton-antiporter in hCMEC/D3 cells (*n* = 4 in triplicate), then incubated for 5 min at 37 °C with KH buffer exempt of [^3^H]-clonidine and containing or not (control) an unlabelled substrate candidate at a range of 0.01 to 1000 µM for clonidine, cocaine, and naloxone experiments, or at 10 µM and/or 100 µM for the other tested candidate substrates. At the end of this second phase the experiment was stopped by placing the cells on ice, removing the incubation buffer, and washing them once rapidly with DPBS. Cells were then lysed with SDS 10% (30 min at 37 °C under agitation). Cell lysates were kept to quantify proteins (micro BCA protein assay kit, Pierce, Sigma). The intracellular tritium radioactivity was measured in a Tri-Carb liquid scintillation counter (Perkin Elmer) after placing and mixing cell lysates in liquid scintillation vials and mixing with 3 mL of Ultima gold XR (Perkin Elmer).

### 2.4. Quantification of the Trans-Stimulation Effect for Selected Substrates

Observed final quantity of intracellular radiolabelled substrate with the log of the various concentration of unlabelled compound (0.01 to 1000 µM) was fitted to a four parameters logistic equation with GraphPad Prism (San Diego, CA, USA); Equation (1):

(1) $$Y=Y_{\max}+\frac{\left(Y_{0}-Y_{\max}\right)}{1+10_{{}_{}}^{log((TSE_{50}-X)*\gamma )}}$$

where *Y* is the percent of intracellular [^3^H]-clonidine compared to control experiment (without unlabelled drug), *X* is the log concentration of unlabelled compound used in the incubation buffer, *Y*_max_ is the maximum effect of trans-stimulation (i.e., minimum level of intracellular [^3^H]-clonidine in percent of control), and *Y*_0_ corresponds to control value without any trans-stimulation compound or when the level of intracellular [^3^H]-clonidine is maximum (≈100%). *TSE*_50_ (µM) is the unlabelled compound concentration at which the trans-stimulation effect is at 50% (or when the intracellular quantity of substrate is equal to *Y_0_*−*Y*_max_/2). Gamma (*γ*) corresponds to the sigmoidal Hill coefficient.

### 2.5. Determination and Classification of Substrate Candidates by [^3^H]-Clonidine Trans-Stimulation Experiments in hCMEC/D3 Cells

The trans-stimulation experiments were also assessed as described above without (control) and with the tested substrate candidate at selected concentrations: 10 and/or 100 µM. Observed final quantity of the intracellular [^3^H]-clonidine with and without (control) the tested candidate was expressed as a mean (Y; %; *n* = 4 in triplicate). According to the Y value measured at 10 µM, the compound was classified as a good substrate (G) and medium-high (M-H) for Y value lower and greater than 50%, respectively, meaning that the TSE_50_ will supposedly be <10 µM for G substrate. The lack of statistical difference between the amount of intracellular [^3^H]-clonidine in control and tested substrate candidate at 10 µM made the experiment at 10 µM inconclusive and led to perform the experiments at 100 µM. Experiments were performed with and without (control) of the tested substrate candidate at 100 µM if needed. According to the Y value reached at 100 µM, the compound was classified medium-low (M-L) substrate (Y < 50%), weak (W) substrate (Y > 50%), or not substrate (N-S) if there was a lack of statistical difference between control [^3^H]-clonidine remaining intracellular amount with and without the tested (100 µM) candidate substrate.

### 2.6. Data Transport Analysis

The data are means ± SD. One-way ANOVA and post hoc test (Dunnett) were used to identify significant differences, unless specified otherwise. Statistical significance was set at *p* < 0.05. The TSE_50_ were estimated using Equation (1) and four parameters logistic fitting with GraphPad Prism software (v7.04, GraphPad, San Diego, CA, USA).

### 2.7. Pharmacophore Generation

Two pharmacophore models for substrates of the proton-antiporter were generated using the FLAPpharm module [23] within the FLAP 2.1 software (Molecular Discovery Ltd., Borehamwood, UK, www.moldiscovery.com, accessed on 1 April 2015) [30]. Since the nature of the chemical structures selected for alignment can influence the pharmacophore model, two datasets selected according to different criteria were used for pharmacophore generation. The first selection for generation of pharmacophore P1 was composed of four well-known proton-antiporter substrates (oxycodone, naloxone, cocaine, and clonidine). Compounds were aligned in their most abundant protonation states as predicted by MoKa 2.5 (Molecular Discovery Ltd., Borehamwood, UK) [31] within the FLAP 2.1 software, and 30 conformers for each structure were generated to perform the alignment. 

The second unbiased selection for generation of pharmacophore P2 was obtained from a Principal Component Analysis (PCA) model generated using compounds in Table 1 as objects and the 128 VolSurf+ chemical-physical and ADME related descriptors of VolSurf+ (Molecular Discovery Ltd., Borehamwood, UK, www.moldiscovery.com, accessed on 20 April 2015) [32] as variables. The most abundant protomeric state at pH 7.4 for each compound was calculated and corresponding chemical structures were imported in VolSurf+. A PCA model built on VolSurf+ descriptors allows exploring the chemical space of the substrates to be explored, to exploit whether or not chemical features can alone define areas in the model related to certain biological effects. To this aim, in this study the objects in the unbiased generated PCA score plot were colored for the experimental substrate effect. The first principal component (PC1) was able to discriminate between substrates and non-substrates, while the second principal component (PC2) highlighted differences among good substrates. The four compounds for selection were obtained by applying the Most Discriminant Compounds (MDC) algorithm available in VolSurf+. No constraints were applied in pharmacophore generation.

### 2.8. Virtual Screening

The FLAP 2.1 software was used to run virtual screenings [30]. A preliminary validation of the pharmacophore models was performed by generating a FLAP database containing the 40 available compounds with known biological effect (Table 1). For each compound, the protonation states at physiological pH were predicted and protomers having a predicted abundance lower than 20% were discarded. A maximum of 50 conformers was generated for each protomer. Virtual screenings were performed using pharmacophores P1 or P2 as templates (“normal” accuracy, “fields” (PIFs) mode). The validation screenings allowed the best descriptor for substrate and non-substrate discrimination to be selected, and for both P1 and P2 it resulted to be the H*O*H descriptor. Afterwards, additional virtual screenings were performed to search for novel substrates, using the Specs database (Specs-SC_20mg_total_Nov2013.sdf from the vendor), the Tropsha’s Human Intestinal Transporter Database [33], the Recon2 (based on MODEL 1109130000 data) [34], and the Human Metabolome Database (HMDB from http://www.hmdb.ca/ accessed on 1 April 2015) [35]. The virtual screenings were performed using P1 or P2 pharmacophore models as templates, following a similar work-flow recently used for the discovery of inhibitors of the proton-antiporter [9]. Briefly, a pre-filtering screening in FLAP bit-string mode [30] was performed as a first step. A molecular weight (MW) filter was applied, processing only compounds with a MW in the range 150–500. A “donor-charged” constraint was also applied, based on the consideration that all the known substrates contained a positively charged donor atom (protonated amine) at physiological pH. At the end of the pre-filtering run, compounds were ranked by Glob-Sum similarity score, and a FLAP database was generated for the 200 top-ranked compounds; for each chemical compound, all protomers with a predicted abundance greater than 20% at pH = 7.4 were imported in a maximum of 50 conformers. Then, a second virtual screening in the GRID-fields mode, as the pharmacophore validation procedures, was performed on the 200 top-ranked compounds using P1 or P2 as templates (settings “normal” accuracy, “fields” (PIFs) mode). At the end of the second virtual-screening, compounds were ranked by their H*O*H molecular similarity score. The selection of compounds for possible testing was obtained by applying cut-off values on the ranking scale. Indeed, for both P1 and P2 the cut-off values were obtained from the H*O*H similarity scores of the validation virtual screening on the 40 compounds in Table 1, being the values immediately higher than the ones for the first top-ranked non-substrate. Thus, for P1 the H*O*H cut-off value was set at 0.19 (the top-ranked non-substrate L-carnitine has a similarity score of 0.18), while for P2 a cut-off value of 0.13 was used (the non-substrate ergothioneine has a similarity score of 0.12). The cut-off values are clearly shown in Tables S2 and S3, in the Supplementary Material. Among the final selected compounds, representative compounds were selected based on chemical diversity and commercial availability criteria. Compounds that are neutral or zwitterionic at pH 7.4 were discarded to increase the probability of substrate identification.

## 3. Results

### 3.1. Characterization of Substrates by Trans-Stimulation Experiments

**Figure 1:**
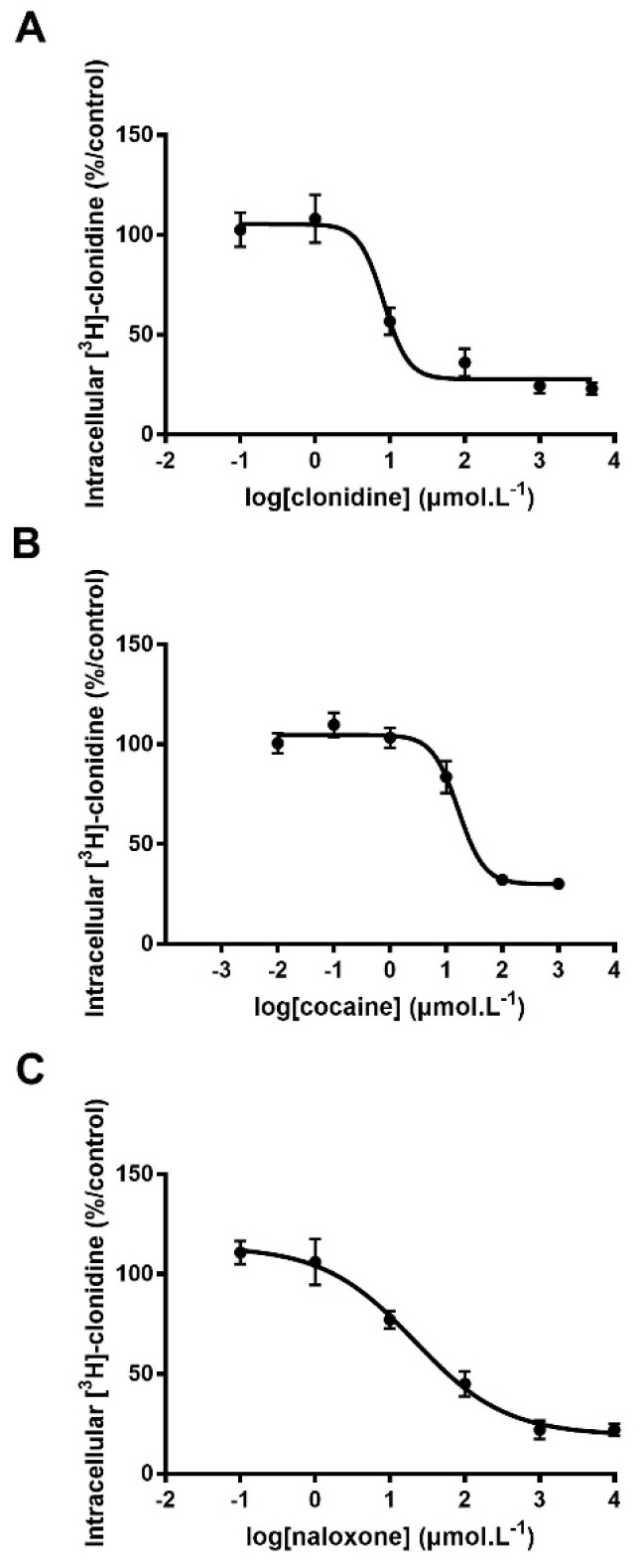
Trans-stimulation studies of [^3^H]-clonidine transport in hCMEC/D3 cells. hCMEC/D3 cells were first loaded with [^3^H]-clonidine for 5 min and then washed and incubated with Krebs-HEPES (KH) buffer alone (control; 100%) or with the unlabeled test compound in KH buffer at 6 concentrations from 0.01 to 1000 µmol·L^−1^. Compounds tested were clonidine (panel **A**), cocaine (panel **B**) and naloxone (panel **C**). Data represent means ± SD of experiments performed in triplicate. The TSE_50_ for clonidine, cocaine, and naloxone in [^3^H]-clonidine trans-stimulation experiments in hCMEC/D3 cells was fitted according to Equation (1).

Clonidine, cocaine and naloxone are known substrates of the proton-antiporter, and their respective intrinsic uptake transport clearance (calculated as the Vmax/Km ratio) measured in hCMEC/D3 cells was 77.3, 34.7, and 15.3 µL·min^−1^·mg^−1^, respectively [9,11]. The TSE_50_ measured in our current experiments was 8.3, 16.5, and 25.9 µM for clonidine, cocaine, and naloxone, respectively (Figure 1). Intrinsic proton-antiporter clearance and TSE_50_ values showed a similar ranking order, with clonidine displaying the highest substrate capacity and naloxone the lowest. These TSE_50_ values prompted us to test two concentrations (10 and 100 µM) for further screening and classification (see Methods) of candidate substrates.

The dataset set of substrates and non-substrates (N-S) (Table 1) used for trans-stimulation experiments and classification was mainly selected according to previous direct demonstrations of their mechanistic role or absence of such a role for this organic cation/proton-antiporter process at the plasma membrane (Table S1). The qualitative classification obtained by trans-stimulation experiments (Table 1) appropriately discriminated between substrates and N-S as expected from their known properties. However, for some drugs classified as substrates, direct evidence of their carrier-mediated transport was still not obtained, as for norbuprenorphine or cocaethylene (Table S1).

### 3.2. Compound Selection for Pharmacophore Generation

**Figure 2:**
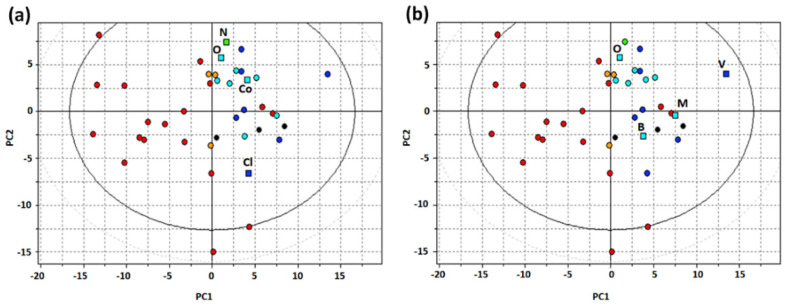
PCA t1-t2 score plot for substrates and non-substrates of the proton-antiporter using VolSurf+ descriptors (G, blue circles; M-H, cyan circles; M-L, green circles; W, orange circles, N-S, red circles): (**a**) Selection of the four substrates used for the generation of pharmacophore P1 (squares: N = naloxone, O = oxycodone, Co = cocaine, Cl = clonidine); (**b**) Selection of the four substrates used for the generation of pharmacophore P2 (squares: O = oxycodone, B = brimonidine, M = methadone, V = verapamil).

In the present study, we generated two pharmacophore models from two sets of active ligands, based on different selection criteria. The first selection (S1) was composed of oxycodone, naloxone, cocaine, and clonidine (Figure S1A). The reason for this selection was that, at the time of this study, they were the best-known substrates for this proton-antiporter [8,10,11,13,20]. The second selection (S2) was unbiased, and generated from a PCA model based on the compounds listed in Table 1, using holistic VolSurf+ descriptors [32]. The MDC algorithm was applied to select the most representative compounds in the series of good (G) and medium-high (M-H) substrates, resulting in the final composition of S2 of oxycodone, methadone, verapamil, and brimonidine (Figure S1B). The score plot of the PCA model is reported in Figure 2, S1 and S2 selections are highlighted in panels A and B, respectively. 

In addition to being an unbiased tool for drug selection, the PCA model obtained provided valuable information on the physicochemical and ADME features of G and M-H substrates. Indeed, the first principal component (PC1) distinguishes between substrates and N-S. Among the VolSurf+ descriptors, as for inhibitors [9], G and M-H substrates showed greater LogP and %FU10 values than N-S. In Volsurf+, the %FU10 descriptor is the fraction of unionized compound at pH 10. In addition, CACO2 and LogBB descriptors, indicating intestinal and BBB permeability, respectively, were also high for G and M-H substrates, suggesting that such drugs pass through membranes readily. Thus, inhibitors and substrates of the proton-antiporter share several common features, such as high lipophilicity and basicity. The second component (PC2) distinguishes between the substrates based on hydrophobicity (see Figure S2).

Comparing selections S1 and S2 in Figure 2, S1 appeared to display worse coverage of the chemical space of substrates, with oxycodone, naloxone and codeine all being located in the same region of the score plot (Figure 2A). Thus, we reasoned that the S2 selection would be more effective in representing the category of substrates, independently of the specific chemical features of the individual molecules.

### 3.3. Generation and Validation of a Pharmacophore Model for Substrates of the Proton-Antiporter

**Figure 3:**
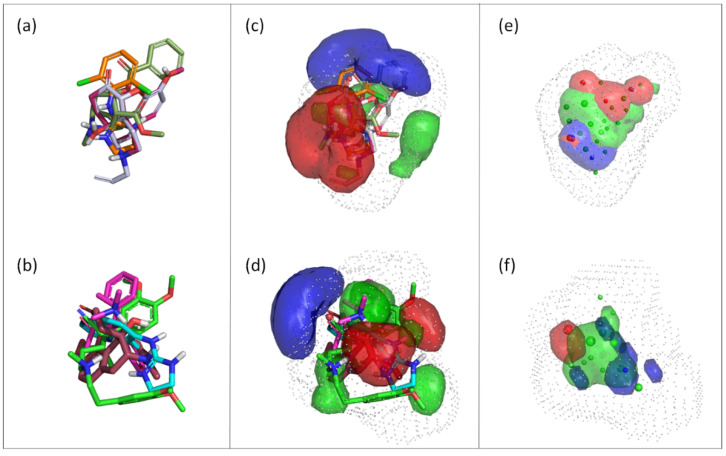
Pharmacophore models for the proton-antiporter substrates generated using FLAP: (**a**) Alignment of the S1 selection and the corresponding pharmacophore model, P1; (**b**) Alignment of the S2 selection and the corresponding pharmacophore model P2. For pharmacophores P1 and P2, oxycodone (dark red) is depicted in the same orientation as the reference.; (**c**) Representation of the FLAPpharm pharmacophore for S1 in the PIFs mode; (**d**) Representation of the FLAPpharm pharmacophore for S2 in the PIFs mode; (**e**) Representation of the FLAPpharm pharmacophore for S1 in the pseudoPIFs mode; (**f**) Representation of the FLAPpharm pharmacophore for S2 in the pseudoPIFs mode.

Compounds of S1 and S2 were aligned by FLAPpharm, and a pharmacophore model for each selection was generated to highlight common features. The highest-scoring pharmacophore models for S1 and S2, named P1 and P2, respectively, are displayed in Figure 3. In particular, once structures are aligned (Figure 3a,b), a FLAPpharm pharmacophore can be represented in terms of these common molecular interaction fields named pharmacophoric interaction fields (PIFs), or in terms of common atom-centered pseudopharmacophoric fields (pseudoPIFs) derived by collapsing original PIFs into the atomic coordinates, as depicted in Figure 3c–f, respectively. More details on the nature of the FLAPpharm pharmacophore and its successful applications have been provided elsewhere [23,25,36,37]. Being based on MIFs, these pharmacophores mimic the potential interactions with the transporter, not in terms of atomic coordinates of the ligand, but in terms of potential molecular recognition. For example, PIFs shown in Figure 3c,d mimic all the regions in which an aminoacidic residue can be located to set the interaction with a given chemical feature of the pharmacophore. One can also notice that comparing a pharmacophore described as PIFs (Figure 3c,d) or pseudoPIFs (Figure 3e,f) colors for H-bond donor and H-bond acceptor features are inverted: this is expected, as in the PIF mode the fields describe how the pharmacophore “feels” the transporter, while in the pseudoPIFs mode the fields describe how the transporter “feels” the pharmacophore. For brevity purposes, the pharmacophores in the pseudoPIFs mode will be briefly described (Figure 3e,f). Pharmacophore P1 was composed of a hydrophobic core (green region and points) between two extended polar regions: an H-bond donor region (blue) and an H-bond acceptor region (red). Pharmacophore P2 was rather similar, but the H-bond donor region (blue) was larger, and the H-bond acceptor region (red) was localized around a single pharmacophore point. The distance between the donor and the acceptor features in P2 (red and blue point in Figure 3f) is 6.1 Å.

**Figure 4:**
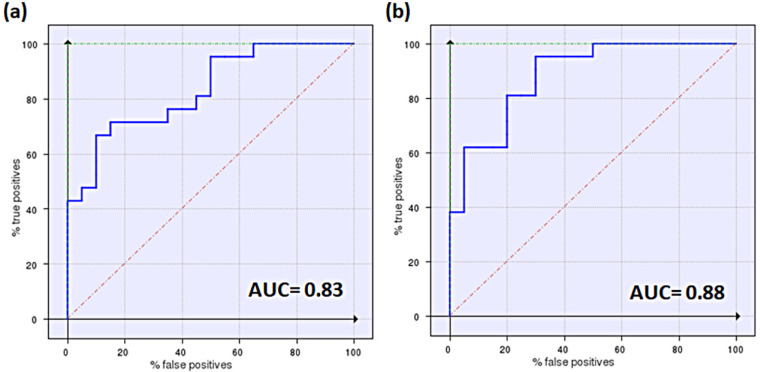
Enrichment plots (ROC curves) for validation of the two pharmacophores, P1 (**a**) and P2 (**b**). AUC values are also reported according to descriptors with the best performances (H*O*H for P1 and P2).

To preliminary validate the two pharmacophore models, they were used as templates for the virtual screening of the original dataset of 40 compounds using FLAP software, to search for the best correlation between the similarity score and the substrate/N-S effect. Among the 18 FLAP descriptors, the best correlation (highest AUC and best early enrichment) was observed for the H*O*H descriptor, generated by a combination of shape (H) and H-bond acceptor (O) features of the MIF fields. Similarity scores are reported in Tables S2 and S3 in the Supplementary Material, while the corresponding ROC curves are shown in Figure 4. Indeed, ROC plots have the advantage of providing a simple visualization of the screening results and a comparison of the discrimination power of the different FLAP descriptors, and can be applied also to small datasets [38]. In terms of AUC values, the use of P2 led to a slightly better performance; however, we decided to run the virtual screening on external databases using both pharmacophore models, to compare results.

### 3.4. Pharmacophore-Based Selection of Possible Novel Substrates by Virtual Screening

One of the most robust strategies to validate a pharmacophore is represented by its usage in virtual screening campaigns followed by experimental tests on the selected hits. Therefore, in the next step, pharmacophore models P1 and P2 were used as templates for large virtual screening campaigns, to search for novel substrates of the proton-antiporter. A first virtual screening was run on a database of commercially available compounds from Specs (www.specs.net, SC_specs_20mg_Nov2013.sdf, accessed on 20 November 2013). In agreement with the validation results, the similarity score calculated for the H*O*H descriptor was used for ranking. To maximize the probability of finding new good (G) substrates, a different cut-off value for compound selection was used for each pharmacophore. Indeed, validation results indicated that the highest ranked known N-S had a similarity score of 0.18 based on P1 (Table S2) and a similarity score of 0.12 based on P2 (Table S3). Thus, the cut-off value for compound selection was set at 0.19 and 0.13 for P1 and P2, respectively (see also Methods). A preliminary observation of compounds in Table 1 indicated that all known substrates were positively charged at physiological pH. Therefore, zwitterionic, neutral, or negatively charged compounds were filtered out. For each pharmacophore, ten top-ranked compounds were selected using similarity score, commercial availability, and visual inspection criteria, avoiding the selection of highly similar compounds. The lists of selected compounds for P1 and P2 are shown in Table 2 and Table 3, respectively. None of the selected compounds in the P1 series was included in the top-ranked compounds of the P2 virtual screening, and vice versa.

A comparison of the compounds selected for P1 and P2 revealed much interesting information. For example, with respect to the protonable moiety, four out of the ten compounds (3, 4, 5, and 8) selected by P1 screening (Table 2) possessed a 4,5-dihydro-1H-imidazole group, which is also present in clonidine. Contrarily, although brimonidine (used for P2 pharmacophore generation) also contains the 4,5-dihydro-1H-imidazole group, none of the compounds selected for P2 (Table 3), presented this chemical feature. This is probably related to the broader chemical variability among S2 compounds than those of S1 (Figure S1).

In addition, it is noteworthy that, although clonidine is classified as a good substrate, compounds 3, 4, 5, and 8 in the P1 series, which share the same 4,5-dihydro-1H-imidazole group, display very diverse biological effects. Indeed, only compound 8 was a G like clonidine, while compound 5 was M-H and compounds 3 and 4 were N-S. 

Similarly, in the P2 series (Table 3) seven out of ten compounds (12–14, 16–19) possessed an aliphatic cyclic amine as a protonable group, and displayed various substrate effects. Thus, although the presence of a net positive charge seems a typical feature of substrates of the proton-antiporter, it is the overall structure of the compound rather than the protonable group that seems to play an important role in interaction with the antiporter.

In addition to this structural information, the performance of the two pharmacophores was also evaluated. The P2 model proved to be more efficient at picking substrates, with 2 G, 4 M-H, and 2 W substrates. Only two compounds (16 and 17) were N-S. In contrast, the P1 model was able to identify only 2 G and 2 M-H substrates, with the rest of the compounds being N-S. Thus, in agreement with the validation data discussed above, according to which P2 was a slightly better model (Figure 4), virtual screening of the Specs database confirmed the superiority of the P2 model over the P1 model.

### 3.5. Additional Screenings on Databases Containing Drugs, Natural and Endogenous Compounds, and Their Evaluation by Trans-Stimulation Assay

The screening performed on the Specs database proved useful for validating the pharmacophores, with P2 performing better than P1. Thus, additional virtual screening was performed on three databases containing compounds known to be drugs or natural or endogenous species. In particular, the Tropsha’s Human Intestinal Transporter Database [33], Recon2 (based on MODEL 1109130000 data [34] and the Human Metabolome Database (HMDB from http://www.hmdb.ca/ accessed on 1 April 2015 [35]) were selected. The first one is a collection of about 3700 unique chemicals known to interact with transport proteins; the second one is a database of more than a thousand human endogenous compounds; the latter is a collection of more than 40,000 human metabolites.

To run the screenings, only compounds in the 150–500 molecular weight range were considered in their most abundant protonation state at pH 7.4. Thus, the above-mentioned pharmacophores P1 and P2 were used as templates. For results’ inspection, the same cut-off values used for Specs database screening to select compounds after ranking were used, and only compounds resulting mostly positively charged at physiological pH according to MoKa prediction were retained, since the observation of known substrates suggests that a positive charge would be crucial to drive the interaction with the antiporter. 

The final lists of selected compounds are provided in Tables S4 and S5. Among them, a further selection was performed for in vitro testing, based on similarity score, commercial availability, and visual inspection criteria. The final dataset tested is shown in Table 4, and their mean Y (%) trans-stimulation value in Figure S3.

Due to the lower cut-off value derived from the validation process, more compounds were retained by the screening performed using P2 as a template. Three compounds (cocaine, hydrocodone and oxycodone) were top-ranked in both virtual screenings and were found to be M-H substrates according to trans-stimulation studies in hCMEC/D3 cells. Among the 23 compounds tested from the P2 virtual screening, 20 were found to be G or M-H substrates, while among the 10 compounds from the P1 virtual screening, only four compounds were classified as G or M-L substrates. Concerning dextromethorphan, this G substrate was identified only for P1 in Table 4; however, its similarity score towards P2 was 0.11, only slightly below the cut-off threshold used in our analysis. Thus, the virtual screening of the additional databases once again confirmed the superiority of the P2 pharmacophore model over the P1 model.

### 3.6. Comparison of the Pharmacophores for Inhibitors and Substrates

Once the best pharmacophore model (P2) for substrates of the proton-antiporter was identified and validated, a comparison with the recently published pharmacophore for inhibitors of the same antiporter was performed [9]. However, it is worth noting that the inhibitors identified, display a competitive mechanism of action. As a result, a large number of good inhibitors were also good substrates (e.g., desomorphine, diphenhydramine, norbuprenorphine, and verapamil), and several weak inhibitors were found to be weak substrates (e.g., morphine and nicotine). On the other hand, this trend is not universal: indeed, clonidine and oxycodone are weak inhibitors but G and M-H substrates, respectively.

**Figure 5:**
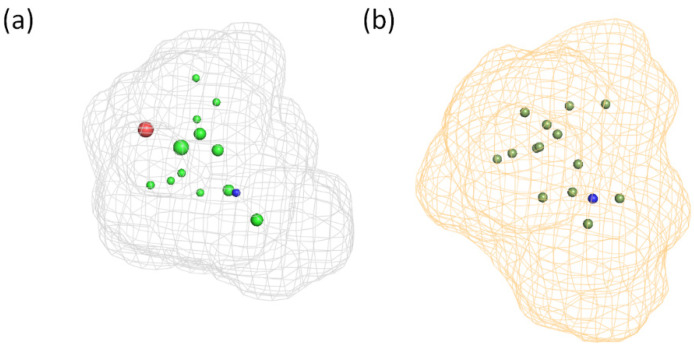
Pharmacophore models for substrates (**a**) (this study) and inhibitors (**b**) from reference [9].

In addition, among the four compounds used to generate the substrate pharmacophore model P2, verapamil and methadone are also good inhibitors, while oxycodone and brimonidine are weak inhibitors. Thus, based on this consideration, the pharmacophore models generated for substrates or inhibitors are expected to be very similar but not identical. For easy comparison, the two pharmacophores are shown in Figure 5. Particularly, the main difference between the inhibitor and substrate pharmacophores relies on the presence of an additional H-bond acceptor point for the substrate P2 model. H-bond acceptor features were also present in the other model for substrate proposed in this study (P1) and thus this feature is likely to be significant. However, the comparison of three-dimensional models is rather difficult by a simple visual inspection, and the best way to evaluate differences is to make a cross-validation.

**Figure 6:**
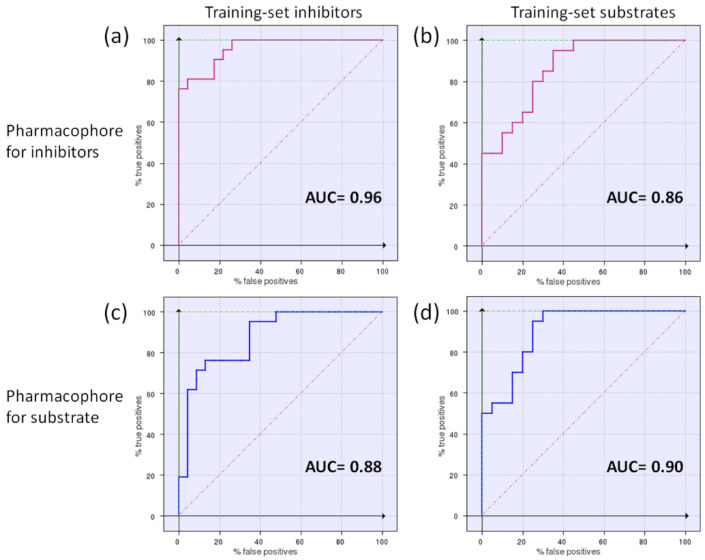
Enrichment plots (ROC curves) obtained by the cross-validation study. The pharmacophore model for inhibitors [9] (**a**,**b**) and the P2 model for substrates (**c**,**d**) were used to screen the datasets for inhibitors or substrates. AUC values are provided, according to the best descriptors for discrimination corresponding to each model: the Glob-Prod descriptor for the inhibitor pharmacophore [9] (**a**,**b**), and the H*O*H descriptor for the substrate pharmacophore (**c**,**d**).

Figure 6 shows the ROC curves obtained screening the datasets for inhibitors and substrates with both pharmacophores in Figure 5. From Figure 6 it emerges that in general the two pharmacophore models perform slightly better when used to screen their related dataset (Figure 6a,d), and the minor differences among the screenings reflect the competitive mechanism of inhibition.

## 4. Discussion

In the present work, a pharmacophore-based approach was applied using FLAPpharm to define the main features of substrates of a previously described polyspecific organic cation/proton-antiporter [8,11,12,19]. The approach was first preliminary validated on 40 known compounds, and then by performing virtual screenings of databases of commercially available compounds, drug databases and collections of natural and endogenous compounds followed by experimental testing on the selected hits, aiming at identifying new substrates. Many psychoactive drugs and known substrates of the proton-antiporter were appropriately identified by the in silico screening, and interestingly, some new compounds were also identified by the optimized pharmacophoric substrate model. This strategy could help in more effectively designing or identifying drugs that could target this BBB proton-antiporter.

Concerning the in silico strategy, two pharmacophore models of the proton-antiporter substrate were developed starting from different sets of compounds for substrate alignment. These two sets of compounds were selected in such a way as to also contain molecules with a weak inhibitory effect but medium-to-good substrate properties. Despite the different sets of compounds used, the two pharmacophores generated shared similar features. Indeed, both of them were composed of a hydrophobic core located between two extended polar regions with opposing features (i.e., an H-bond donor and an H-bond acceptor); although, minor differences were detected. The pharmacophore model generated from verapamil, oxycodone, brimonidine, and methadone, which were selected by an unbiased PCA analysis to improve chemical variability, (named P2) displayed the best performance in terms of the identification of novel substrates both among commercially available compounds from the Specs vendor, and among known drugs and endogenous compounds. When the P1 pharmacophore model, generated from naloxone, oxycodone, cocaine and clonidine, was used to screen the Specs database, there was less chemical variability among the top-ranked compounds, with four out of ten hits bearing the 4,5-dihydro-1H-imidazole moiety. This lack of diversity not only confirmed the superiority of P2 over P1 in picking substrates with different scaffolds, but also revealed that the presence of the 4,5-dihydro-1H-imidazole moiety, which is present in two good substrates (clonidine and brimonidine), does not per se indicate substrates of this proton-antiporter. The information derived by the nature and by the application of pharmacophore P2 may provide first knowledge on the design of novel substrates. For example, in addition to the need of a positive charge, the distance of about 6 Å between the acceptor region and the main donor region in the ligand coordinates may be helpful in delimiting the chemical space of ideal substrates. In addition, we demonstrated that the nature of protonable nitrogens in good substrates is highly variable, including tertiary amines, 4,5-dihydro-1H-imidazole, and pyridines. Finally, rigidity appears a key feature to be taken into account. The FLAP software used here for virtual screening purposes also allows compounds around the FLAPpharm pharmacophores to be designated, and thus will result in useful information for future studies.

Many compounds selected from the above mentioned virtual screening are known to be psychoactive drugs, implying that they “cross the BBB”. Drug distribution to the brain can be evaluated by a partition parameter, K_p,brain,uu_, calculated as the ratio of the amount of unbound drug in the brain to that in the blood at a steady state kinetics. K_p,brain,uu_ could thus provide a global information about the predominant role of import carrier-mediated transport across the BBB if it is greater than one, whereas a value below one would be related more to the predominant export of the drug out of the brain [39]. However, this parameter, whatever its value, could never rule out the simultaneous but quantitatively dissimilar contributions of both export and import processes at the BBB. Studies using a large range of CNS-active drugs have established that some are actually substrates of P-gp (e.g., K_p,brain,uu_ ~0.2 for risperidone [40]), which definitively rules out the concept that P-gp substrates are CNS-inactive drugs [41]. Although the export of drugs from the brain has mainly been illustrated as involving the P-gp transporter, the role of BBB drug importers for CNS drugs has been far less documented.

Some psychoactive drugs, such as antidepressant compounds from the tricyclic class (e.g., doxepin, imipramine, and amitriptyline) or antipsychotics from the phenothiazine class (e.g., chlorpromazine), have been shown to exhibit K_p,brain,uu_ values greater than two in rodents, suggesting the role of a carrier-mediated BBB importer [40]. Our experiments suggest that the drug/proton-antiporter is involved in the import of these compounds/substrates. Some related drugs from the tricyclic and phenothiazine classes were also predicted to be proton-antiporter substrates by our current virtual screening and in vitro study (e.g., promazine, and desipramine). However, their K_p,brain,uu_ values have not been reported, to our knowledge, and the quantitative contribution of each diffusion component (e.g., passive and carrier-mediated transport) still needs to be assessed. Likewise, our pharmacophore model and in vitro tests helped to classify some antihistamine-H1 drugs with known CNS sedative properties as substrates of this proton-antiporter: promethazine (with a phenothiazine core), pheniramine, chlorpheniramine, diphenhydramine, and triprolidine. Indeed, the K_p,brain,uu_ of diphenhydramine has been shown to be greater than two in many species including sheep, rodents, dogs, and non-human primates, and its property as a proton-antiporter substrate has been clearly demonstrated at the BBB and brain-retinal barrier in rats and mice [10,16,20,42]. Some opiates and opioids such as oxycodone, oxymorphone, dextromethorphan, hydrocodone, and methadone have also emerged as substrates of the proton-antiporter. Oxycodone is already known to be a substrate and the others have been suggested to be substrates by microdialysis or in situ brain perfusion experiments (e.g., oxymorphone and methadone) [13,20,43]. D617, a verapamil metabolite, was predicted by virtual screening to be a proton-antiporter substrate in the present study. Similar to verapamil, D617 probably lacks a CNS target, but a PET study using [^11^C]-D617 has evidenced its significant distribution in the brain parenchyma [44]. 

The role of simultaneous BBB import and export processes for several drugs has also been illustrated in previous studies using rodent models lacking P-gp function [18]. In summary, two situations have been identified depending on which process is dominant: net efflux or net influx. Compounds with a K_p,brain,uu_ lower than 1 (e.g., 0.2 for the antipsychotic drug risperidone) suggest a predominant role of drug efflux. In P-gp-deficient mice, the K_p,brain,uu_ can be increased above 1, as shown for risperidone, which reaches a K_p,brain,uu_ of 2.3 [40], also pointing to the role of an importer in addition to the predominant P-gp-mediated export masking the effect of the importer when both transporters are expressed at the BBB. This has also been illustrated with verapamil, for which P-gp transport dominates overall BBB transport, masking the involvement of the proton-antiporter influx at the mouse BBB, unless revealed by the lack of P-gp [18]. The opposite situation can also be illustrated with venlafaxine, an antidepressant unrelated to the tricyclic drugs, which exhibits a K_p,brain,uu_ of 3.5 in rodents [40]. This suggests a predominant import process at the BBB, which is in accordance with our experiments predicting that venlafaxine is a proton-antiporter substrate.

## 5. Conclusions

In conclusion, the pharmacophore model P2 presented here for substrates of the BBB proton-antiporter could help to better predict drug interaction with the transporter and to design new chemical entities with enhanced brain influx transport. However, such optimization should also take into account at least the possible effects of P-gp/BCRP efflux, which could counterbalance any changes to the BBB transport rate. This study underlines the critical role of this drug/proton-antiporter in the carrier-mediated BBB transport for many psychoactive drugs into the brain, and expands our knowledge about the properties of this critical SLC transporter at the BBB.

## Figures and Tables

**Table 1:** Dataset of substrates and non-substrates.

Compound	Classification ^(a)^	Compound	Classification ^(a)^
Clonidine	G	Morphine	W
Desomorphine	G	Nicotine	W
Diphenhydramine	G	Agmatine	N-S
Heroine	G	Choline	N-S
Norbuprenorphine	G	Cimetidine	N-S
Tramadol	G	Dihydromorphine	N-S
Verapamil	G	Dopamine	N-S
6-monoacetylmorphine	M-H	Ergothioneine	N-S
Brimonidine	M-H	Guanidine	N-S
Cocaethylene	M-H	Histamine	N-S
Cocaine	M-H	L-dopa	N-S
Codeine	M-H	L-carnitine	N-S
Methadone	M-H	Melatonin	N-S
Norcocaine	M-H	Milnacipran	N-S
Oxycodone	M-H	MPP	N-S
Pyrilamine	M-H	N-methylnaloxone	N-S
Apomorphine	M-H	Paraquat	N-S
MDMA	M-H	Serotonin	N-S
Naloxone	M-L	Tetraethylammonium	N-S
Hydromorphone	W	Tyramine	N-S

^(a)^ G (good substrate), M-H (medium-high substrate), M-L (medium-low substrate), W (weak substrate) and N-S (non-substrate): classification as per in vitro trans-stimulation studies in hCMEC/D3 cells. MPP, 1-methyl-4-phenylpyridinium; MDMA, methylenedioxy-methylamphetamine.

**Table 2:** List of 10 substrate candidates for the proton-antiporter, based on virtual screening results using the P1 pharmacophore as a template and classified by their substrate potency in hCMEC/D3 cells.

ID	Specs Compound Code	Structure	Similarity-Score ^(a)^	Classification ^(b)^
1	AI-204/34841050	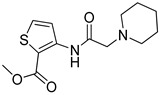	0.41	N-S
2	AO-476/43407062	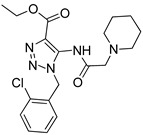	0.39	N-S
3	AE-641/00584045	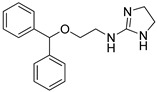	0.38	N-S
4	AE-641/00605007	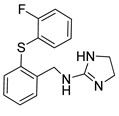	0.37	N-S
5	AE-641/11703012	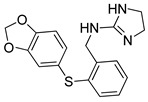	0.37	M-H
6	AN-329/43448732	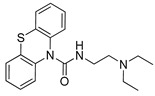	0.36	G
7	AE-562/12222547	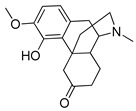	0.35	M-H
8	AE-641/00191008	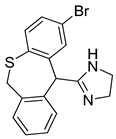	0.34	G
9	AK-968/10817037	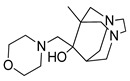	0.33	N-S
10	AE-641/30156016	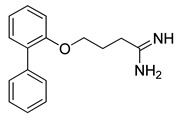	0.29	N-S

^(**a**)^ Similarity score generated by FLAP 2.1 software for the H*O*H descriptor. ^(**b**)^ Compounds were classified by in vitro trans-stimulation studies in hCMEC/D3 cells as G (good substrate), M-H (medium-high substrate), M-L (medium-low substrate), W (weak substrate), and N-S (non-substrate).

**Table 3:** List of 10 substrate candidates for the proton-antiporter, based on virtual screening results using the P2 pharmacophore as a template and classified by their substrate potency in hCMEC/D3 cells.

ID	Specs Compound Code	Structure	Similarity-Score ^(a)^	Classification ^(b)^
11	AE-641/00335028	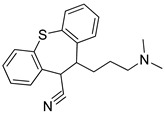	0.21	G
12	AE-907/30533025	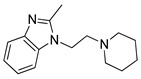	0.21	M-H
13	AO-476/43023620	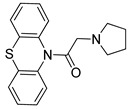	0.21	M-H
14	AO-365/41690614	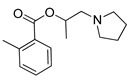	0.19	M-H
15	AN-465/41588344	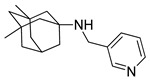	0.18	G
16	AG-205/14365199	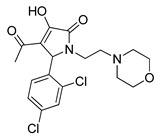	0.17	N-S
17	AO-365/43401585	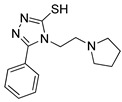	0.17	N-S
18	AP-906/42717100	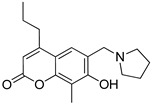	0.17	W
19	AF-399/37418022	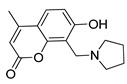	0.14	W
20	AI-942/13331402	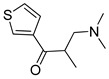	0.14	M-H

^(**a**)^ Similarity score generated by FLAP 2.1 software for descriptor H*O*H. ^(**b**)^ Compounds were classified by in vitro trans-stimulation studies in hCMEC/D3 cells as G (good substrate), M-H (medium-high substrate), M-L (medium-low substrate), W (weak substrate) and N-S (non-substrate).

**Table 4:** List of substrate candidates for the proton-antiporter based on the virtual screening results of drug and natural/endogenous compound databases using either P1 or P2 as templates and in vitro substrate classification by trans-stimulation studies in hCMEC/D3 cells.

Substrate Candidate	Database	Pharmacophore	Classification
Amitriptyline	A	P2	G
Buflomedil	A	P2	G
Chlorpheniramine	A	P2	G
Chlorpromazine	A	P2	G
Cocaine	B	P1, P2	M-H
Desipramine	A	P2	G
Dextromethorphan	A	P1	G
Diphenhydramine	C	P2	G
Doxapram	A	P2	M-L
Doxepin	A, C	P2	G
Hydrocodone	C	P1, P2	M-H
Hydromorphone	C	P1	W
Hydroxyl-melatonin	B	P2	N-S
Imipramine	A	P2	G
Mecamylamine	C	P2	G
Methadone	A, C	P2	M-H
Methixene	A	P1, P2	G
Nalbuphine	C	P1	W
Naloxone	C	P1	M-L
Oxycodone	C	P1, P2	M-H
Oxymorphone	C	P1	M-L
Pheniramine	A	P2	M-H
Promazine	A	P2	G
Promethazine	A	P2	G
Sibutramine	C	P2	G
Trihexyphenidyl	A	P2	G
Triprolidine	C	P2	G
Venlafaxine	A	P1, P2	M-L
D617-verapamil metabolite	A	P2	M-L

The databases used are A: Tropsha, B: Recon 2, and C: HMDB. Two pharmacophores for substrates P1 and P2 were established using a diverse set of substrates and used to screen the databases. Compounds were classified by in vitro trans-stimulation studies in hCMEC/D3 cells as G (good substrate), M-H (medium-high substrate), M-L (medium-low substrate), W (weak substrate) and N-S (non-substrate).

## Data Availability

Data of the study are available from the corresponding author upon reasonable request.

## Supplementary Materials

The following are available online at https://www.mdpi.com/article/10.3390/pharmaceutics14020255/s1, Figure S1: Chemical structures of drugs used for the generation of pharmacophore P1(a) and P2 (b), Figure S2: Loading plot for the PCA model generated for substrates and non-substrates of the proton antiporter, Figure S3: Substrate candidates Y (%) measured at 10 or 100 µM by the [^3^H]-clonidine trans-stimulation experiments in hCMEC/D3 cells. Table S1: List of compounds classified as substrate or non-substrate of the proton-antiporter according to direct evaluation, Table S2: Dataset used for the P1 pharmacophore validation and virtual screening based on the FLAP HOH similarity score descriptor, Table S3: Dataset used for pharmacophore P2 validation and virtual screening based on the FLAP HOH similarity score descriptor, Table S4: Substrate candidates after virtual screening on the Tropsha’s database (A), the Recon2 Database (B), and the HMDB database (C), using pharmacophore P1 as a template, Table S5: Substrate candidates after virtual screening on the Tropsha’s database (A), the Recon2 Database (B) and the HMDB database (C), using pharmacophore P2 as a template.
