# Supplementary material for: Pharmacophore-Based Discovery of Substrates of a Novel Drug/Proton-Antiporter in the Human Brain Endothelial hCMEC/D3 Cell Line
Source: Pharmaceutics. 2022 Jan 21;14(2):255. doi: 10.3390/pharmaceutics14020255 (PMC8875908; doi:10.3390/pharmaceutics14020255)
Supplement: Supplementary file 1 [file pharmaceutics-14-00255-s001.zip › pharmaceutics-1534177-supplementary.pdf]

pharmaceutics

# Supplementary Materials : Pharmacophore-Based Discovery of Substrates of a Novel Drug/Proton-Antiporter in the Human Brain Endothelial hCMEC/D3 Cell Line

**Maria Smirnova, Laura Goracci, Gabriele Cruciani, Laetitia Federici, Xavier Declèves, Hélène Chapy, and Salvatore Cisternino**

## A. Supplementary Tables

**Table S1.** List of compounds classified as substrate or non-substrate of the proton-antiporter according to direct evaluation (SUB, Substrate; N-S, Non-substrate; ND, Not documented;* our unpublished data).

| Substrate* | Status | Non Substrate* | Status |
|---|---|---|---|
| Clonidine | SUB[1,2] | Morphine | ND |
| Desomorphine | ND | Nicotine | SUB [5] |
| Diphenhydramine | SUB[3,4] | Agmatine | N-S[14] |
| Heroine | ND | Choline | N-S [15] |
| Norbuprenorphine | ND | Cimetidine | N-S [16] |
| Tramadol | SUB [5,6] | Dihydromorphine | ND |
| Verapamil | SUB [7] | Dopamine | N-S [17] |
| 6-monoacetylmorphine | ND | Ergothioneine | N-S [18] |
| Brimonidine | SUB [8] | Guanidine | N-S [19] |
| Cocaethylene | ND | Histamine | N-S [17] |
| Cocaine | SUB [9] | L-dopa | N-S [20] |
| Codeine | SUB [10] | L-carnitine | N-S [21] |
| Methadone | ND | Melatonin | ND |
| Norcocaine | ND | Milnacipran | N-S * |
| Oxycodone | SUB [11] | MPP | N-S [17] |
| Pyrilamine | SUB [11] | N-methylnaloxone | ND |
| Apomorphine | SUB [12] | Paraquat | N-S [22, 23] |
| MDMA | SUB [13] | Serotonin | N-S [17] |
| Naloxone | SUB [2] | Tetraethylammonium | N-S * |
| Hydromorphone | ND | Tyramine | ND |

[1](Andre et al., 2009), [2](Chapy et al., 2015), [3](Auvity et al., 2016), [4](Sadiq et al., 2011), [5](Cisternino et al., 2013), [6](Kitamura et al., 2014), [7](Chapy et al., 2016), [8](Zhang et al., 2006), [9](Chapy et al., 2014), [10](Fischer et al., 2010), [11](Okura et al., 2008), [12](Okura et al., 2013), [13](Kuwayama et al., 2008), [14](Wang et al., 2014), [15](Allen and Smith, 2001), [16](Murakami et al., 2000), [17](Andre et al., 2012), [18](Shimizu et al., 2015), [19](Al-Khawaja et al., 2014), [20](Kageyama et al., 2000), [21](Berezowski et al., 2004), [22] (Bartlett et al., 2011), [23](Vilas-Boas et al., 2014).

**Table S2.** Dataset used for the P1 pharmacophore validation and virtual screening based on the FLAP HOH similarity score descriptor**.**

| Compound | Classification | Similarity Score | Compound | Classification | Similarity Score |
|---|---|---|---|---|---|
| Heroine | G | 0.23 | N-methylnaloxone | N-S | 0.15 |
| Tramadol | G | 0.23 | Brimodinine | M-H | 0.14 |
| Cocaethylene | M-H | 0.23 | Cimetidine | N-S | 0.14 |
| Cocaine | M-H | 0.23 | ergothioneine | N-S | 0.14 |
| Codeine | M-H | 0.22 | Dihydromorphine | N-S | 0.13 |
| Norcocaine | M-H | 0.22 | Verapamil | G | 0.12 |
| Methadone | M-H | 0.21 | L-dopa | N-S | 0.12 |
| Desomorphine | G | 0.2 | Clonidine | G | 0.11 |
| Diphenhydramine | G | 0.2 | Norbuprenorphine | G | 0.11 |
| Hydromorphone | W | 0.19 | Apomorphine | M-H | 0.1 |
| Pyrilamine | M-H | 0.19 | Choline | N-S | 0.1 |
| MDMA | M-H | 0.18 | Serotonin | N-S | 0.1 |
| L-carnitine | N-S | 0.18 | Dopamine | N-S | 0.09 |
| 6-Monoacetylmorphine | M-H | 0.17 | Tyramine | N-S | 0.08 |
| Oxycodone | M-H | 0.17 | Agmatine | N-S | 0.07 |
| Naxolone | M-L | 0.17 | Histamine | N-S | 0.07 |
| Morphine | W | 0.17 | MPP | N-S | 0.03 |
| Nicotine | W | 0.17 | Paraquat | N-S | 0.03 |
| Melatonin | N-S | 0.16 | TEA | N-S | 0.03 |
| Milnacipran | N-S | 0.16 | Guanidine | N-S | 0 |

**Table S3.** Dataset used for pharmacophore P2 validation and virtual screening based on the FLAP H*O*H similarity score descriptor.

| Compound | Classification | Similarity Score | Compound | Classification | Similarity Score |
|---|---|---|---|---|---|
| Methadone | M-H | 0.19 | Clonidine | G | 0.09 |
| Cocaethylene | M-H | 0.18 | Desomorphine | G | 0.09 |
| Heroine | G | 0.17 | Brimodinine | M-H | 0.09 |
| Diphenhydramine | G | 0.16 | L-carnitine | N-S | 0.09 |
| Pyrilamine | M-H | 0.16 | Norbuprenorphine | G | 0.08 |
| Cocaine | M-H | 0.15 | Cimetidine | N-S | 0.08 |
| Naxolone | M-L | 0.15 | Milnacipran | N-S | 0.08 |
| Apomorphine | M-H | 0.15 | Morphine | W | 0.07 |
| Verapamil | G | 0.14 | Dihydromorphine | N-S | 0.07 |
| 6-monoacetylmorphine | M-H | 0.13 | Choline | N-S | 0.06 |
| Oxycodone | M-H | 0.12 | L-dopa | N-S | 0.05 |
| Ergothioneine | N-S | 0.12 | Serotonin | N-S | 0.05 |
| Hydromorphone | W | 0.11 | Agmatine | N-S | 0.04 |
| N-methylnaloxone | N-S | 0.11 | Dopamine | N-S | 0.04 |
| Tramadol | G | 0.1 | Tyramine | N-S | 0.04 |
| Codeine | M-H | 0.1 | Histamine | N-S | 0.03 |
| Norcocaine | M-H | 0.1 | MPP | N-S | 0.03 |
| Nicotine | W | 0.1 | Paraquat | N-S | 0.03 |
| MDMA | M-H | 0.1 | TEA | N-S | 0.03 |
| Melatonin | N-S | 0.1 | Guanidine | N-S | 0 |

**Table S4.** Substrate candidates after virtual screening on the Tropsha's database (A), the Recon2 Database (B) and the HMDB database (C), using pharmacophore P1 as a template.

| Candidate | SMILES | Similarity score* |
|---|---|---|
| Database (A) | | |
| Methixene | CN1CCCC(CC2C3=CC=CC=C3SC3=CC=CC=C23)C1 | 0.23 |
| Dextromethorphan | [H][C@]12CC3=C(C=C(OC)C=C3)[C@@]3(CCCC[C@]13[H])CCN2C | 0.23 |
| Laudanosine-6′-bromo | COc1c(OC)cc(C[C@@H]2[NH+](C)CCc3cc(OC)c(OC)cc23)cc1 | 0.22 |
| Cilazaprilat | C1C[C@@H](C(=O)N2[C@@H](CCCN2C1)C(=O)O)N[C@@H](CCC3=CC=CC=C3)C(=O)O | 0.22 |
| Benactyzine | CC[NH+](CC)CCOC(=O)C(O)(c1ccccc1)c1ccccc1 | 0.20 |
| CHEMBL131459 | CC(C)N(C[C@H](O)COc1c(C(=O)c2ccccc2)c(C)nn1C)C(C)C | 0.20 |
| Venlafaxine | OC1([C@H](c2ccc(OC)cc2)C[NH+](C)C)CCCCC1 | 0.19 |
| N-[2-(3,4-dimethoxyphenyl)ethyl]-1-methylpyrrolidin-2-imine | COc1ccc(CC/[NH+]=C/2CCCN2C)cc1OC | 0.19 |
| CHEMBL339825 | CC(C)N(C[C@H](O)COc1c(C(=O)c2ccccc2)c(C)nn1c1ccccc1)C(C)C | 0.19 |
| Oxybutynin | O[C@](C1CCCCC1)(C(=O)OCC#CCN(CC)CC)c1ccccc1 | 0.19 |
| Database (B) | | |
| Cocaine | C[NH+]1[C@H]2CC[C@@H]1[C@H]([C@@H](C2)OC(=O)c1ccccc1)C(=O)OC | 0.26 |
| (R)-4-phosphopantothenate | CC(C)(CO)C(/C(=N/CCC(=O)[O-])O)O | 0.20 |
| N-Acetylmethionine | [H][C@@](CCSC)(NC(C)=O)C(O)=O | 0.20 |
| Database (C) | | |
| Oxycodone | [NH2+]([C@]1(C([C@@H]2C[C@H]1CC2)(C)C)C)C | 0.38 |
| Oxymorphone | O1[C@@H]2[C@]34[C@](O)([C@H]([NH+](CC3)C)Cc3c4c1c(O)cc3)CCC2=O | 0.31 |
| Naltrexone | O1[C@@H]2[C@]34[C@](O)([C@H]([NH+](CC3)CC3CC3)Cc3c4c1c(O)cc3)CCC2=O | 0.27 |
| Hydrocodone | O1[C@@H]2[C@]34[C@H]([C@H]([NH+](CC3)C)Cc3c4c1c(OC)cc3)CCC2=O | 0.26 |
| Naloxone | O1[C@@H]2[C@]34[C@](O)([C@H](N(CC3)CC=C)Cc3c4c1c(O)cc3)CCC2=O | 0.26 |
| Thebaine | C1[NH+](C)[C@H]2Cc3ccc(c4c3[C@]3(C2=CC=C([C@H]3O4)OC)C1)OC | 0.23 |
| 6-O-Methylcodeine | O1[C@@H]2[C@H](C=C[C@H]3[C@@]42CC[NH+](C)[C@@H]3Cc2c4c1c(OC)cc2)OC | 0.22 |
| Nalbuphine | c12c3c(c(cc1)O)O[C@H]1[C@H](CC[C@]4([C@H]([NH+](CC[C@]314)CC1CCC1)C2)O)O | 0.21 |
| Norbutorphanol | C1CC[C@]2([C@H]3Cc4ccc(cc4[C@]2(C1)CC[NH2+]3)O)O | 0.20 |
| Butorphanol | O[C@@]12[C@]3(CC[NH+]([C@@H]1Cc1c3cc(O)cc1)CC1CCC1)CCCC2 | 0.19 |
| Hydromorphone | O1[C@@H]2[C@]34[C@H]([C@H]([NH+](CC3)C)Cc3c4c1c(O)cc3)CCC2=O | 0.19 |

*According to the FLAP H*O*H descriptor

**Table S5.** Substrate candidates after virtual screening on the Tropsha's database (A), the Recon2 Database (B) and the HMDB database (C), using pharmacophore P2 as a template.

| Candidate | SMILES | Similarity Score* |
|---|---|---|
| Database (A) | | |
| Trihexyphenidyl | O[C@](CC[NH+]1CCCCC1)(C1CCCCC1)[C@H-]1[CH-][CH-][CH+][CH+][CH+]1 | 0.23 |
| Doxylamine | O(CC[NH+](C)C)[C@](c1ncccc1)(c1ccccc1)C | 0.22 |
| Thenyldiamine | C[NH+](C)CCN(Cc1cscc1)c1ncccc1 | 0.19 |
| Methadone | O=C(C(c1ccccc1)(c1ccccc1)C[C@H]([NH+](C)C)C)CC | 0.19 |
| D-617(metabolite of Verapamil) | CC(C)[C@](C#N)(c1cc(OC)c(OC)cc1)CCCNC | 0.19 |
| Doxapram | O=C1N(CC)C[C@@H](CC[NH+]2CCOCC2)[C@@]1([C-]1[CH-][CH-][CH-][CH-][CH-]1)[CH+]1[CH+][CH+][CH+][CH+][CH+]1 | 0.19 |
| Chlorpromazine | Clc1cc2N(c3c(Sc2cc1)cccc3)CCC[NH+](C)C | 0.18 |
| Antazoline | N1CC[NH+]=C1CN(Cc1ccccc1)c1ccccc1 | 0.18 |
| Methixene | C[NH+]1CCC[C@H](C[C@@H]2[C@@H-]3[CH-][CH-][CH-][CH-][C@@H-]3S[C@@H+]3[C@H+]2[CH+][CH+][CH+][CH+]3)C1 | 0.17 |
| Zolamine | C[NH+](C)CCN(Cc1ccc(OC)cc1)c1nccs1 | 0.17 |
| Methapyrilene | C[NH+](C)CCN(Cc1cccs1)c1ccccn1 | 0.17 |
| Fenipramide | C1CC[NH+](CC1)CCC(C(=O)N)(c1ccccc1)c1ccccc1 | 0.17 |
| Gamfexine | C[NH+](C)CC[C@H](C1CCCCC1)c1ccccc1 | 0.16 |
| Laudanosine-6*f*-bromo | COc1c(OC)cc(C[C@@H]2[NH+](C)CCc3cc(OC)c(OC)cc23)cc1 | 0.16 |
| Pheniramine | n1ccccc1[C@@H](c1ccccc1)CC[NH+](C)C | 0.16 |
| Imipramine | c1cc2c(cc1)CCc1c(cccc1)N2CCC[NH+](C)C | 0.16 |
| Promazine | S1c2ccccc2N(c2c1cccc2)CCC[NH+](C)C | 0.16 |
| Procyclidine | O[C@](c1ccccc1)(CC[NH+]1CCCC1)C1CCCCC1 | 0.16 |
| Buflomedil | COc1cc(OC)c(C(=O)CCC[NH+]2CCCC2)c(OC)c1 | 0.16 |
| Amitriptyline | c1cc2c(/C(=C/CC[NH+](C)C)c3c(cccc3)CC2)cc1 | 0.16 |
| Ungerine | CO[C@H]1C=C2CC[NH+](C)[C@H]2[C@H]2[C@@H]1OC(=O)c1cc3c(OCO3)cc21 | 0.15 |
| Levophacetoperane | CC(=O)O[C@@H]([C@H]1CCCC[NH2+]1)c1ccccc1 | 0.15 |
| Chlorpheniramine | Clc1ccc(cc1)[C@@H](c1ncccc1)CC[NH+](C)C | 0.15 |
| ChemSpiderID23281049 | O=C1C[C@H](C[NH+]2CCCCC2)Oc2ccccc12 | 0.15 |
| Isopropamide | NC(=O)C(CC[NH+](C(C)C)C(C)C)(c1ccccc1)c1ccccc1 | 0.15 |
| Xylometazoline | N1CC[NH+]=C1Cc1c(C)cc(cc1C)C(C)(C)C | 0.15 |
| Delcorine | O[C@@H]1[C@H]2[C@@]34[C@H]5C[C@@H]6[C@@H](OC)[C@H]5[C@]5(C[C@@H]6OC)OCO[C@@]15[C@@H]3N(CC)C[C@]2(COC)CC[C@@H]4OC | 0.15 |
| Protriptyline | c1cc2c(C=Cc3c(cccc3)C2CCC[NH2+]C)cc | 0.14 |
| NSC-664565 | [NH+]1=C(NCC1)CN1[C@H](C)[C@H](C)C=C(C)c2ccccc12 | 0.14 |
| Vincamine | O[C@]1(C[C@@]2(CC)CCC[NH+]3CCc4c([C@@H]23)n1c1ccccc41)C(=O)OC | 0.14 |
| Doxepin | O1c2ccccc2/C(=C/CC[NH+](C)C)c2c(cccc2)C1 | 0.14 |
| Eburnamonine | CC[C@@]12CCC[NH+]3CCc4c([C@H]13)n(c1ccccc41)C(=O)C2 | 0.14 |

| | | |
|---|---|---|
| Desipramine | c1cc2c(cc1)CCc1c(cccc1)N2CCC[NH2+]C | 0.14 |
| Promethazine | S1c2ccccc2N(c2c1cccc2)C[C@@H]([NH+](C)C)C | 0.13 |
| Aminopentamide | C[C@H](CC(C(=O)N)(c1ccccc1)c1ccccc1)[NH+](C)C | 0.13 |
| Disopyramide | NC(=O)[C@](c1ccccn1)(c1ccccc1)CC[NH+](C(C)C)C(C)C | 0.13 |
| Oxymetazoline | Oc1c(cc(C)c(CC2=[NH+]CCN2)c1C)C(C)(C)C | 0.13 |
| Maprotiline | CNCCCC12CCC(C3=CC=CC=C13)C1=CC=CC=C21 | 0.13 |
| Database (B) | | |
| Cocaine | C[NH+]1[C@H]2CC[C@@H]1[C@H]([C@@H](C2)OC(=O)c1ccccc1)C(=O)OC | 0.14 |
| 6-Hydroxymelatonin | C/C(=N/CCc1c[nH]c2cc(c(cc12)OC)O)O | 0.14 |
| Database (C) | | |
| Methadone | O=C(C(C[C@@H]([NH+](C)C)C)(c1ccccc1)c1ccccc1)CC | 0.20 |
| Norpropoxyphene | CCC(=O)O[C@@](Cc1ccccc1)([C@@H](C)C[NH2+]C)c1ccccc1 | 0.19 |
| Hydromorphone-3-sulphate | C[NH+]1CC[C@]23[C@@H]4Oc5c(OS(=O)(=O)[O-])ccc(C[C@@H]1[C@H]2CCC4=O)c35 | 0.18 |
| noracymethadol | CC[C@H](C(C[C@@H](C)[NH2+]C)(c1ccccc1)c1ccccc1)OC(=O)C | 0.17 |
| Isothipendyl | S1c2c(N(C[C@@H]([NH+](C)C)C)c3ncccc13)cccc2 | 0.17 |
| Nor-Levomethadyl acetate | CC[C@@H](C(C[C@@H](C)[NH2+]C)(c1ccccc1)c1ccccc1)OC(=O)C | 0.16 |
| Ritalinic acid | c1ccc(cc1)[C@@H]([C@H]1CCCC[NH2+]1)C(=O)[O-] | 0.16 |
| Triprolidine | [NH+]1(CCCC1)C/C=C(/c1ccc(cc1)C)c1ncccc1 | 0.16 |
| Hydrocodone | O1[C@@H]2[C@]34[C@H]([C@H]([NH+](CC3)C)Cc3c4c1c(OC)cc3)CCC2=O | 0.15 |
| (R)-Laudanidine | CN1CCc2cc(c(cc2[C@@H]1Cc1ccc(c(c1)O)OC)OC)OC | 0.15 |
| Sibutramine | Clc1ccc(C2(CCC2)[C@@H]([NH+](C)C)CC(C)C)cc1 | 0.15 |
| Diphenhydramine | C(OCC[NH+](C)C)(c1ccccc1)c1ccccc1 | 0.14 |
| Propylhexedrine | C[C@H](CC1CCCCC1)[NH2+]C | 0.14 |
| Amdinocillin | [C@@H]1([C@@H]2N(C1=O)[C@H](C(S2)(C)C)C(=O)[O-])/[NH+]=C/N1CCCCCC1 | 0.14 |
| Doxepin | C1(=C\CC[NH+](C)C)/c2c(COc3c1cccc3)cccc2 | 0.14 |
| Oxycodone | O1[C@@H]2[C@]34[C@](O)([C@H](N(CC3)C)Cc3c4c1c(OC)cc3)CCC2=O | 0.14 |
| Mecamylamine | [NH2+]([C@]1(C([C@@H]2C[C@H]1CC2)(C)C)C)C | 0.14 |
| 3-Isothiocyanatomethyl-1-methoxy-1H-indole | COn1cc(C[NH+]=C=S)c2ccccc12 | 0.13 |
| 3-Methoxymorphinan | [C@@]123c4c(C[C@@H]([C@H]1CCCC3)[NH2+]CC2)ccc(c4)OC | 0.13 |

*According to the FLAP H*O*H descriptor

**B. Supplementary Figure**

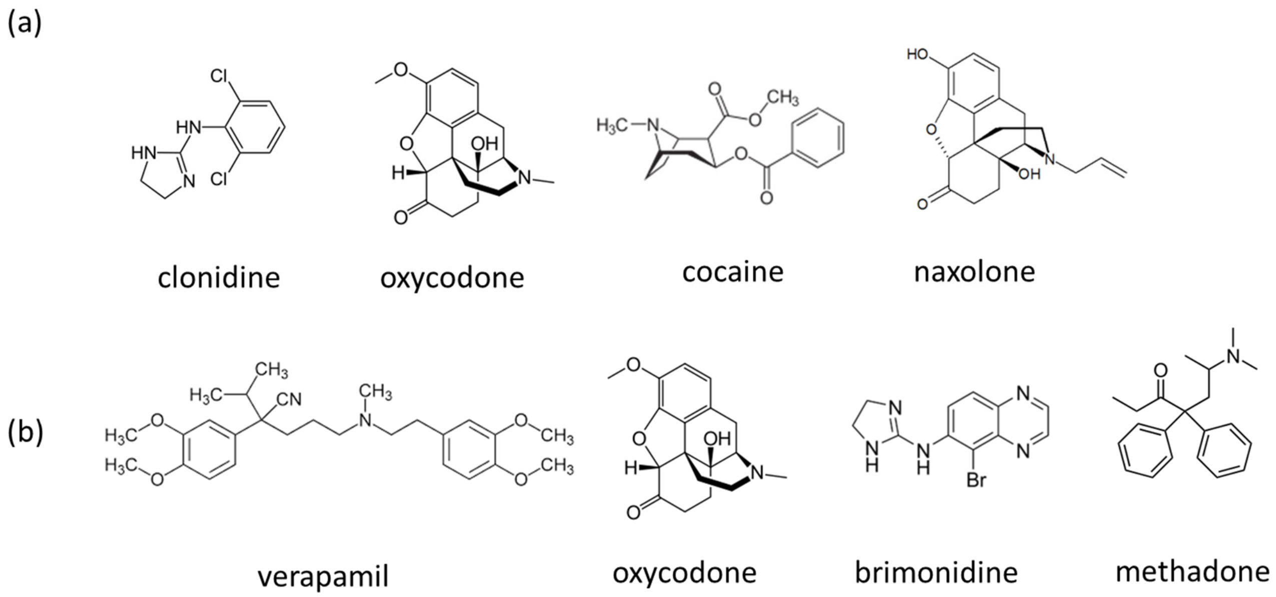


**Figure S1***:* Chemical structures of drugs used for the generation of pharmacophore P1(**a**) and P2 (**b**).

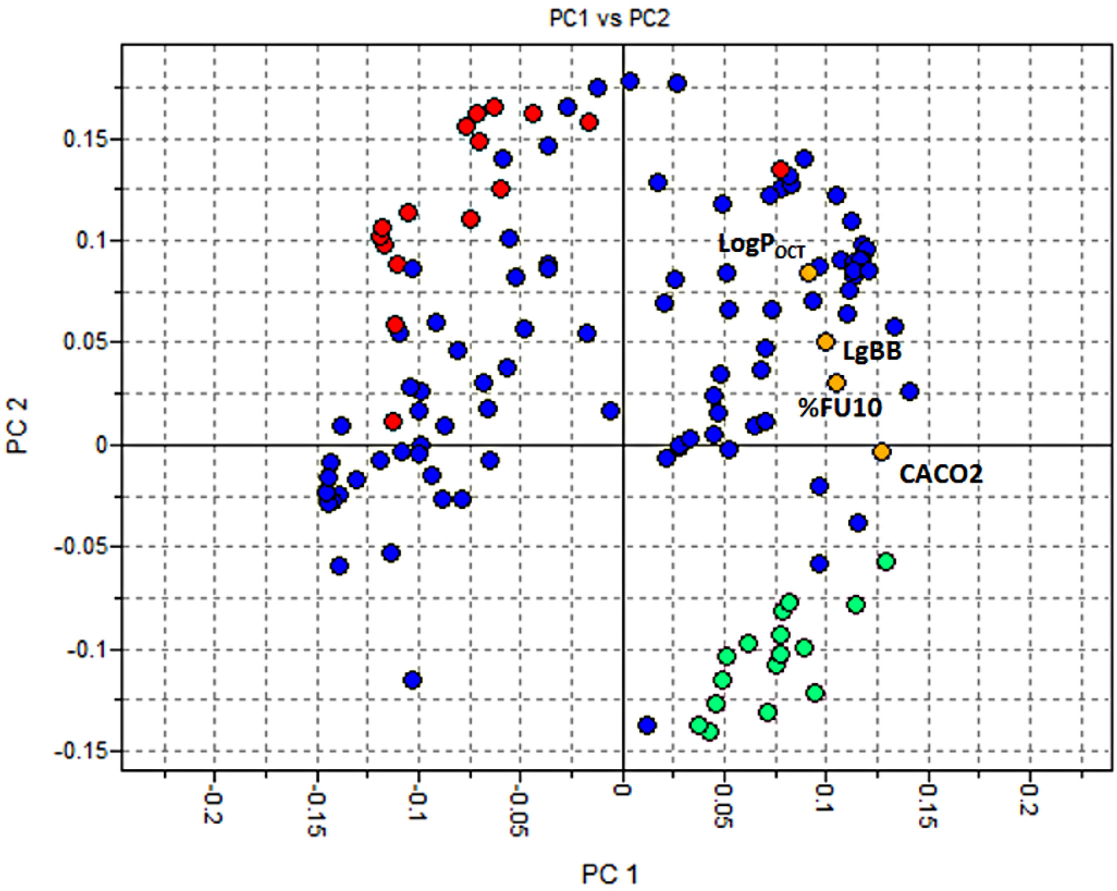


**Figure S2:** Loading plot for the PCA model generated for substrates and non-substrates of the proton antiporter. Hydrophilic descriptors (W1-W8, CW1-CW8) are red colored, while hydrophobic descriptors (D1-D8, CD1-CD8) are green colored. Additional descriptors discussed in the manuscript are highlighted in orange color. The other VolSurf+ descriptors are shown in blue. A detailed description of the descriptors has been reported elsewhere (Chapy et al., 2015).

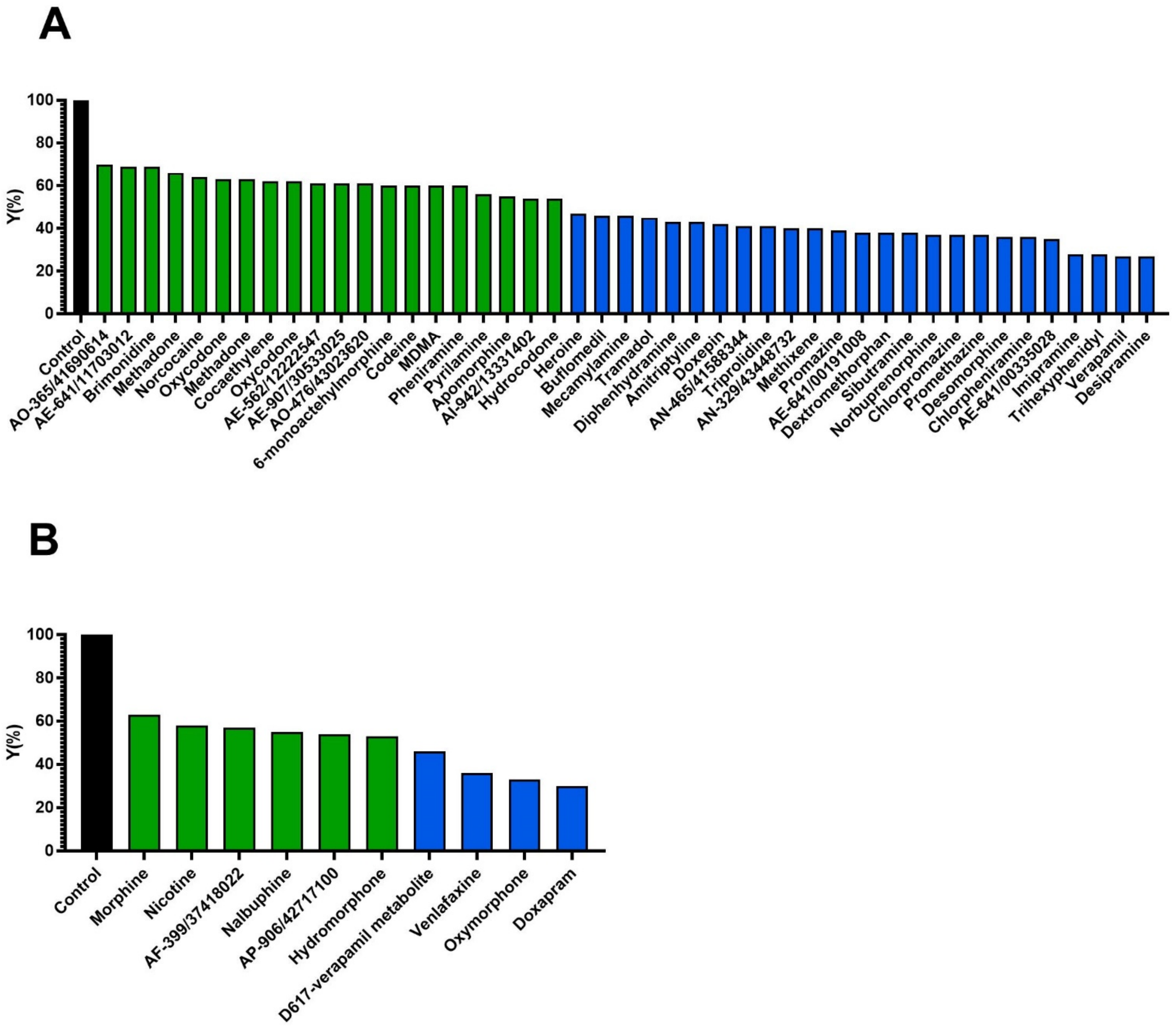


**Figure S3:** Substrate candidates Y(%) measured at 10 or 100μM by the [3H]-clonidine trans-stimulation experiments in hCMEC/D3 cells; (**A**) Y is the percent of intracellular [3H]-clonidine compared to control experiment (without unlabelled drug) obtained with subtrate candidate at 10 μM (blue, "Good" substrate; green, "Medium-high" substrate); (**B**) Y is the percent of intracellular [3H]-clonidine compared to control experiment (without unlabelled drug) obtained with substrate at 100 μM (blue, "Weak" substrate; green, "Medium-low" substrate).
